# Craniocerebral Magnetic Resonance Imaging Features of Benign Paroxysmal Positional Vertigo under Artificial Intelligence Algorithm and the Correlation with Cerebrovascular Disease

**DOI:** 10.1155/2022/8952355

**Published:** 2022-04-26

**Authors:** Hailong Xue, Yanli Jing, Yingge Chen, Cong Xi, Na Bian, Yujuan Li

**Affiliations:** ^1^Department of Neurology, The 987th Hospital of the Joint Logistics Support Force of the People's Liberation Army, Baoji 721004, Shaanxi, China; ^2^Department of Neurology, Baoji City People's Hospital, Baoji 721000, Shaanxi, China

## Abstract

This research aimed to discuss the characteristics of benign paroxysmal positional vertigo (BPPV) and the correlation with cerebrovascular disease. An artificial intelligence algorithm under a parallel dual-domain concatenated convolutional neural network (PDDC-CNN) was proposed to process the images of magnetic resonance imaging (MRI). MRI, magnetic resonance angiography (MRA), and susceptibility weighted imaging (SWI) were performed on all 60 research objects with a 3.0 MRI scanner. The number of cases with cerebral microbleeds (CMBs), SWI image display of small veins, the number of lacunar infarctions, vertebral artery dominance, and vertebrobasilar morphology were observed in the two groups. The number of lacunar infarctions was 2.400 ± 3.358 and 0.672 ± 0.251, respectively, in the BBPV group with 30 cases and the control group with the other 30 cases. The positive rates of CMBs on SWI images were 48% and 27% in the BBPV group and the control group, respectively, and the average CMBs were counted as 1.670 ± 2.326 and 0.487 ± 0.865. CMBs were shown as round or oval lesions of conventional sequence deletion in the images with a diameter of less than 1.5 cm. SWI images of the BBPV group showed a significant increase in intracerebral small veins compared to those of the control group. The curvature of the vertebrobasilar artery in the BBPV group was significantly higher than that in the control group, and the curvature of the basilar artery was slightly higher than that in the control group. In conclusion, the MRI features of BPPV patients were related to their own microvascular lesions closely, and it was speculated that the cerebrovascular factors might play a dominant role in the early onset of BPPV.

## 1. Introduction

Vertigo is a common symptom in emergency with various causes. Benign paroxysmal positional vertigo (BPPV) is one of the most common vertigoes clinically. It is more common in people over 60 years old, and about 30% of people over 70 years old have experienced it at least once in their lives. The symptoms of BBPV are rare in children and are classified as peripheral vestibular syndrome [1]. BPPV is caused by the rapid movement of relative gravity at a certain position of the head, accompanied with paroxysmal vertigo of short-positioning-angle-rotation linear nystagmus and relatively short time [2]. Its main clinical symptoms are the short-term rotational nystagmus caused by rapid head movement in a specific position, where the vertical line angle of relative gravity had a large change [3–5]. It is believed that BPPV is in a close relation to various vascular factors. These factors can cause serious damage to the blood vessels of the inner ear directly and may lead to the shedding of otoliths, which are likely to be the causes of BPPV [6]. Cerebral microbleeds (CMBs) are a kind of preclinical changes in microvascular fibrous hyaline change and hemosiderin deposits caused by slight extravasation of blood. It is also called old CMBs, type II lacunar hemorrhage, punctate hemorrhage, and so on [7]. The symptoms of CMBs patients are relatively insidious. However, as the patients' condition continues to develop, it may also cause more obvious early bleeding. As a potential bleeding disease state, CMBs are related to the sequelae of acute cerebrovascular disease and the decline of cognitive system function, and they can be taken as a marker in early disease screening [8]. Therefore, it is of great clinical value for the early diagnosis of CMBs.

In recent years, deep learning has made great breakthroughs in magnetic resonance imaging (MRI) reconstruction and has shown broad prospects. It has received extensive attention from researchers and has become one of the popular research methods [9, 10]. Convolutional neural networks have unique advantages in image processing. Compared with stacked autoencoders, deep confidence networks, and deep Boltzmann machines that can only input vectors, they can extract features and learn nonlinear mapping relationships [11]. The success of the early convolutional neural network AlexNet had attracted the attention of scholars; for the first time, Cao et al. [12] applied this classic deep learning network to compressed sensing MRI. The development of digital clinical medical imaging diagnosis technology in the 21st century has led to the technological advancement of modern clinical medicine in disease treatment and follow-up diagnosis. Thus, clinical imaging diagnostic medicine takes a leading position in the diagnosis of most clinical diseases [13]. The etiology of BPPV is complex, and it is difficult to be diagnosed and classified. The pathogenesis of BPPV is not yet clear, and research is mostly focused on the treatment of the disease. In addition, there is insufficient clinical understanding, which makes it difficult to diagnose correctly. There is little research on BPPV in imaging.

Susceptibility weighted imaging (SWI) is an emerging technology that has become more and more widely used in MRI contrast enhancement in recent years. Its biggest feature is that it can detect hemorrhages more sensitively than conventional MRI sequences, especially for microhemorrhages or punctate hemorrhages. The working principle of SWI vibration imaging lies in the highly differentiated imaging and the dependent effect of oxygen content in tissue blood, which are caused by the non-uniform magnetic field of the partial tissues. In intracranial vascular imaging, phase contrast (PC) and time-of-flight (TOF) methods are very sensitive to blood flow velocity and intracranial vascular distribution, and it is difficult to accurately distinguish between arteries and veins. It would not be limited by the many imaging conditions mentioned above when SWI is used, which can only present a clear visualization of intracranial venous vessels. Thereby, it has great technical advantages for intracranial venous vessel observation and vascular malformation detection [14].

In summary, how to diagnose BPPV accurately is a major issue that needs to be paid attention to in clinical practice. There are a number of research results showing that the treatment of BPPV is in connection with the local vascular motion factors. The conventional 3.0 t laser MRI scanning technology was used and combined with susceptibility and weighted MRI technology, to study in depth the correlation between intracranial local vascular movement factors and BPPV. It was expected to provide new help for the etiology, treatment, and early prevention of BPPV through the new perspective of clinical imaging.

## 2. Materials and Methods

### 2.1. Research Objects

Thirty patients with BPPV were selected, who were diagnosed in the hospital from early November 2019 to March 2020.12 males and 18 females were included, aged 20–68 years old with an average age of 41.37 ± 10.17 years. People who underwent normal physical examination without vertigo symptoms during the same period were selected as the control group, and a total of 30 cases were chosen. All patients signed an informed consent form, and this study had been approved by the ethics committee of the hospital.

Inclusion criteria: patients were diagnosed with BPPV through routine examinations. They agreed to cooperate with the hospital treatment and regular follow-up actively. They had a history of recurrent short-onset vertigo.

Exclusion criteria: patients took vestibular inhibitory drugs, antihistamines, and other anticholinergic drugs recently. They had a history of trauma, vascular diseases, metabolic diseases, or more. The patients failed to cooperate with hospitalization and long-term follow-ups.

### 2.2. Network Model Design

**Figure 1:**
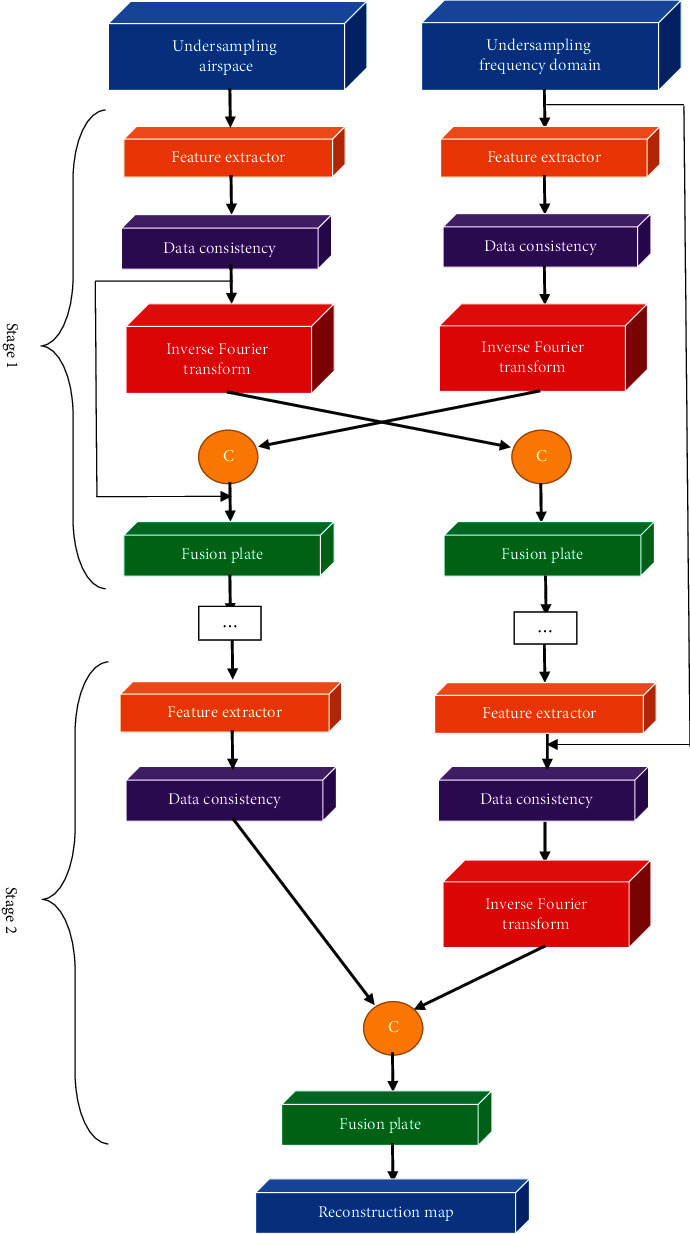
PDDC-CNN under deep learning and compressed sensing. The direction of the arrows indicated the direction of data flow.

The new parallel dual-domain concatenated convolutional neural network (PDDC-CNN) was proposed under deep learning and compressed sensing. The overall structure of the network is shown in Figure 1.

The feature extractor and fusion module used in different domains had the same structure, but they did not share parameters. The parameters were not shared among the same modules in different stages. After the undersampling frequency domain and undersampling spatial data passed through the feature extractor and the data consistency layer, there was a cross-fusion process to fuse the feature maps from the two domains into those in one domain. The conversion from domain to domain was performed using Fourier forward and inverse transform.

The undersampling frequency domain and undersampling spatial domain data were used as input, and the reconstructed image was the output. The dual data streams were not only parallel but also cross-fused. The whole process was divided into five stages, which were five concatenated networks. Stages 1–4 had the same structure exactly, which were mainly used for artifact reduction and detail restoration. Stage 5 was to output the final reconstructed image, so the input and output of the fusion module were slightly different.

With the upgrading of hardware conditions, issues such as increased calculation, overfitting, gradient disappearance, and gradient explosion gradually emerged, when the network structure of the current deep learning model was deeper with more convolutional layers. The performance of image reconstruction reached saturation and was unable to continue to improve. Therefore, the residual was introduced into the convolutional neural network. The output of the previous layer of the convolutional neural network and the output of the last layer were added through a direct connection line. This connection neither increased the parameters of the network nor increased the complexity of the calculation. The basic residual network was used to express, where *a*_*m*_ was the observed value, *a*_*m*+1_ was the predicted value, *K*_*a*_*m*_,*c*_*m*__ was the residual, and *c*_*m*_ represented the mapping matrix. The residual network consisted of two parts, with *g*(*·*) representing the direct mapping part. The residual part corresponded to the convolution part, and it could be expressed as

(1) $$\begin{array}{c} a_{m+1}=g\left(a_{m}\right)+K_{a_{m},c_{m}}. \end{array}$$

In the multilayer residual structure, the predicted value of the *M* layer could be obtained from the observation value of any layer *l* shallower than it plus the sum of the residuals between them. It was expressed as

(2) $$\begin{array}{c} a_{M}=a_{m}\sum_{h=1}^{M-1}K_{a_{m},c_{m}}. \end{array}$$

It was supposed that the loss function was *θ*, to find the derivative with respect to *a*_*m*_. The chain derivative equation was expressed as the

(3) $$\begin{array}{c} \frac{\partial_{\theta }}{\partial_{a_{m}}}=\frac{\partial_{\theta }}{\partial_{a_{M}}}\frac{\partial_{a_{M}}}{\partial_{a_{m}}}=\frac{\partial_{\theta }}{\partial_{a_{M}}}\left(1+\frac{\partial }{\partial_{a_{m}}}\right)\sum_{h=m}^{M-1}K_{a_{m},c_{m}}. \end{array}$$

During the entire training process, the residual network would not have a gradient disappearance.

### 2.3. Image Examination and Image Processing

A superconducting 3.0MR automatic scanner was used, with a standard cranial 8-channel automatic scanning coil. The 60 objects underwent T1 weighted imaging (T1W1), T2 weighted imaging (T2W1), diffusion weighted imaging (DWI), T2-fluid attenuated inversion recovery (FLAIR), TOF, MRA, and SWI sequence scanning in cranial transverse axis. The imaging parameters included sensitivity encoding (SENSE), and three-dimensional T1 fast field echo (FFE). Time of repetition (TR) of 17.0 ms, time of echo (TE) of 25.0 ms, reversal angle of 15°, a layer thickness of 2 mm, no interval acquisition, a field of view (FOV) of 230 × 183 × 100 mm, matrix of 256 × 203, number of excitations of 1.0, and scanning time of 2 minutes and 38 seconds were also included.

SWI can produce the magnitude image (MI) and the phase image (PI) at the same time through one scanning. For obtaining the SWI image, the original MI and PI need to be transmitted to the workstation for processing. The PI was filtered by appropriate frequency, and 32 × 32 or 64 × 64 high-pass filter matrix was selected to filter out background low-frequency artifacts and retain the benefits of high-pass filtering. A high-pass filter map, the corrected PI, was obtained. The corrected PI and MI were integrated, and the SWI map was obtained through minimum density reconstruction.

### 2.4. Judgment of Cerebral Lacunar Infarction

MRI lacunar infarction was manifested as lesions in the bilateral subcortices with a diameter of less than 15 mm and clear boundaries with long T1 and long T2 signals. It was different from the enlargement of the perivascular Virchow–Robin spaces. It was common in the white matter, basal ganglia region, and their surrounding internal capsules of the brain. The total number of intracranial lacunar infarctions in each patient was counted.

### 2.5. CMBs Examination

**Figure 2:**
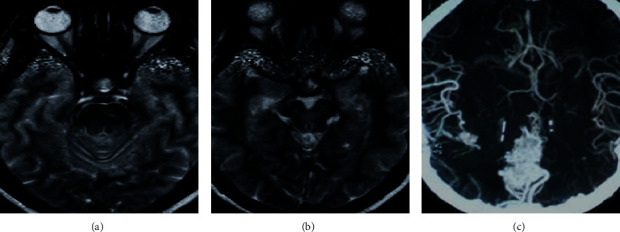
MI and SWI images of CMBs. The arrows pointed out CMBs. (a) The MI image. (b) The SWI image showing low-density signals. (c) The SWI image with clearer intracranial veins processed by the minimum density projection technology.

The MI and SWI images showed low signal areas similar to an ellipse uniformly distributed on CMBs, which were not completely connected to the previous or next layer. The diameter was about 1.5–6.5 mm, and there was no obvious edema in the surrounding tissues. Intracranial calcifications and small blood vessel flow empty shadow should be eliminated in time. Intracranial calcifications were usually symmetrical, irregular in shape, and common in choroid plexus, globus pallidus, etc. Small blood vessel flow empty shadow was more common in the cerebral cortex area and was usually shown as short T2WI signals. By tracing the course of the blood vessels in the continuous layers, it could be distinguished.  Figure 2 shows the images of CMBs.

### 2.6. Vertebrobasilar Artery Information

The measurement method of blood vessel width and diameter was described as follows. The sequential convergence point of the vertebrobasilar arteries was the starting point of the detection. The width of the bilateral vertebral arteries was measured with three points separated by 0.3 cm, and the average of their widths was taken as the measured value of the vertebral artery diameter standard. The following criterion was for judging the vertebral artery dominance. The difference in the diameter of the bilateral vertebral arteries was ≥0.3 mm, or was not much different in the image, and it was relatively closely connected with the basilar artery. According to the difference in the diameter of the bilateral vertebrobasilar arteries, it was divided into three grades. Grade I showed a difference between the bilateral artery diameters of 0.04–0.70 mm, Grade II had a difference of 0.71–1.17 mm, and Grade III had a difference of 1.18–2.67 mm.

A straight line was drawn between the junction of the bilateral vertebral basilar artery and the beginning of the basilar artery. This straight line was also called basilar artery length (BAL), which was the length standard line of the basilar artery. The bending length (BL) referred to the average distance from the most bending point of the basilar artery to the BAL. The types of abnormal vertebrobasilar curvature mainly included the C type, reverse C type, and S type.

### 2.7. Statistical Methods

The data analysis and processing were performed via Spss19.0. The measurement data were expressed as mean ± standard deviation. The comparisons between the measurement group and the control group were performed through an independent sample *t*-test. The ranked data were tested by the rank sum difference method. The chi-square test or Fisher's exact probability method was adopted to express the enumeration data. The inspection level of data analysis results was *α* = 0.05.

## 3. Results

### 3.1. Statistical Results of Lacunar Infarctions

**Figure 3:**
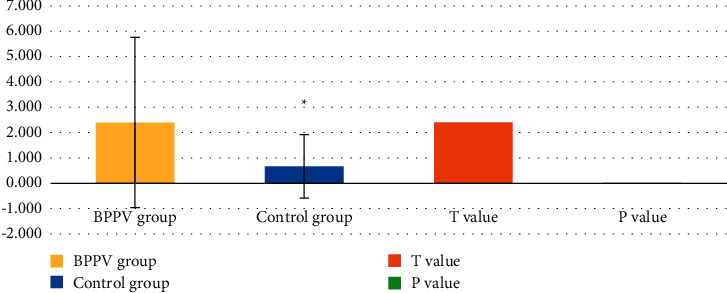
Comparison of the number of lacunar infarctions in the BPPV group and the control group. ^*∗*^The difference was statistically significant compared with the result of the BPPV group (*P* < 0.05).

The average number of lacunar infarctions in the BPPV group was (2.400 ± 3.358), and that in the control group was (0.672 ± 1.252). As shown in Figure 3, the number of lacunar infarctions in the BPPV group was greater than that in the control group (*t* = 2.410, *P* < 0.05).

### 3.2. The Positive Rate of CMBs

**Figure 4:**
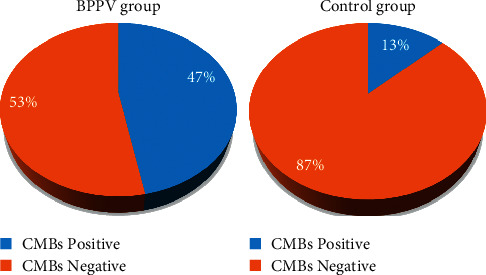
Comparison of the positive rate of CMBs between the BPPV group and the control group.

Among the 30 cases in the BPPV group, 14 cases (47%) were positive for CMBs; and among the 30 cases in the control group, 4 cases (13%) were detected positive for CMBs. The chi-square test was used to statistically analyze the positive rate of CMBs in both groups, and the results are shown in Figure 4.

The results of the positive rate and negative rate of CMBs were analyzed. It was found that the positive rate of CMBs in the BPPV group was higher than that of the control group (*χ*^2^ = 3.309, *P* < 0.05). Among the 18 positive cases, there were at most 10 and at least 1 CMBs lesion. The average number of CMBs in the BPPV group was (1.632 ± 3.017), while the number in the control group was (0.384 ± 0.898). There was statistical significance in the number of CMBs in both groups (*t* = 1.982, *P* < 0.05).

### 3.3. Comparison of the Detection Rate of CMBs by Conventional MRI and SWI Sequence

**Figure 5:**
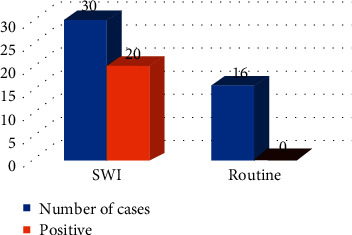
Comparison of the detection rate of CMBs by conventional MRI and SWI sequence.

Among 60 examiners, 20 cases with CMBs were detected by SWI. 40 cases with CMBs were detected by conventional unenhanced MRI, which is shown in Figure 5.

**Figure 6:**
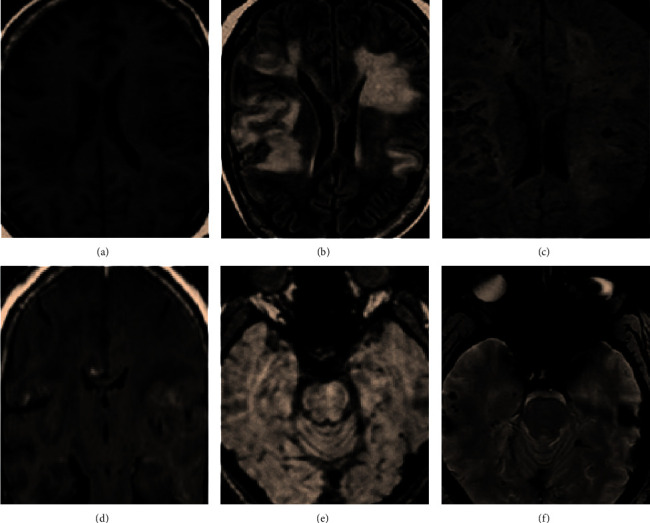
MI and SWI images of CMBs. (a–d) The image of T1WI, T2WI, T2-FLAIR, and DWI (*b* = 1000); these conventional MRI images showed no abnormality. (e) MI and (f) SWI image showed low-signal CMBs.

Both the MI and SWI images of CMBs showed low-signal lesions with clear edges, as shown in Figure 6.

### 3.4. Correlation between Vertebral Artery Dominance as well as Basilar Artery Curvature and BBPV

**Figure 7:**
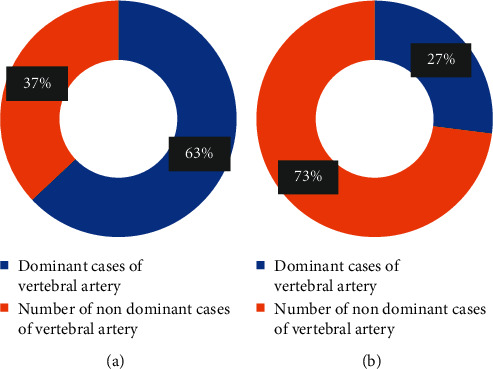
Comparative observation of the vertebral artery dominance in a total of 27 cases in the BPPV group (a) and the control group (b).

**Figure 8:**
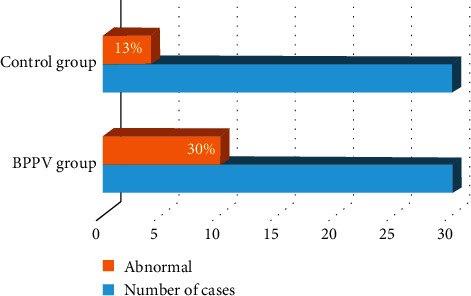
Morphological analyses of the vertebrobasilar artery.

**Figure 9:**
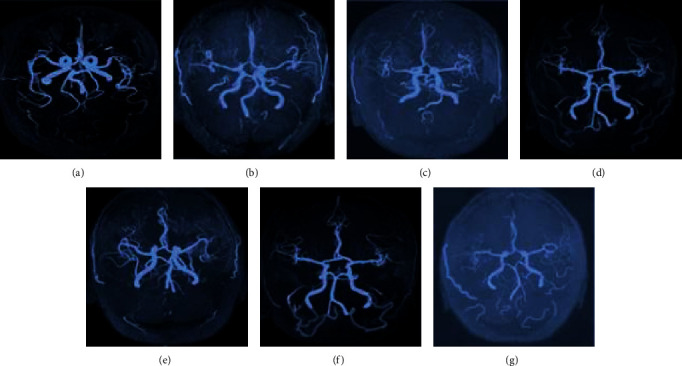
Basilar artery morphology. (a–g) The C type, S type, reverse C type, normal vertebral artery dominance, normality, left side dominance, and right side dominance.

MRA images of 60 objects were measured. It was shown that there were 19 cases with vertebral artery dominance in the BPPV group, among which 10 cases with left side dominance, 9 cases with right side dominance, and 10 cases with basilar artery morphological abnormalities. In the control group, there were 8 cases with vertebral artery dominance, including 3 cases on the left side, 5 cases on the right side; 4 cases with abnormal basilar artery morphology, and the differences were statistically significant (*P* < 0.05). The comparisons of the dominance of the vertebral artery and the basilar artery curvature are shown in Figures 7–9.

## 4. Discussion

BPPV is a kind of motor hallucination or spatial image realization dislocation caused by head position changes. In recent years, some studies in China have paid great attention to the serious impact of cerebrovascular risk factors in BPPV, especially in elderly patients with cerebrovascular diseases. There is a certain correlation between BPPV and cerebrovascular incident factors [15]. High blood pressure and diabetes may cause damage to the capillaries of the inner ear to a certain degree, leading to otolith shedding; it may directly participate in the pathogenesis of BPPV [16]. 30 patients with BPPV were selected as the research objects, and 39 patients without vertigo symptoms who received normal physical examination during the same period were chosen as the control group. All these patients underwent MRI scanning under the PDDC-CNN model. It was found that the average number of lacunar infarctions was (2.400 ± 3.358) in the BPPV group, and was (0.672 ± 1.252) in the control group, showing a remarkable difference between the two groups (*t* = 2.410, *P* < 0.05). This indicated that the cerebral lacunar infarction in BPPV patients was more severe than that in normal objects. The positive rate of CMBs in the BPPV group was higher than that in the control group significantly (*X*^2^ = 3.309, *P* > 0.05), indicating that the incidence of a cerebral hemorrhage in BPPV patients was higher than that in normal people. Thus, it was speculated that the incidence of cerebral hemorrhage was related to the conditions of BPPV. The results of this work could also explain most of the concurrence of cerebral vascular risk factors and BPPV [17].

In the BPPV group, there were 19 cases of vertebral artery dominance, consisting of 10 cases of left dominance and 9 cases of right dominance. There were also 10 cases of abnormal basilar artery morphology. In the control group, 8 cases got vertebral artery dominance, with left dominance in 3 cases and right dominance in 5 cases; 4 cases got abnormal basilar artery morphology. The differences were of statistical significance (*P* < 0.05). Previous research has shown that cerebral small vessel diseases can directly damage the tiny arteries of the inner ear in patients, leading to microcirculation disturbance [18, 19]. This result suggested that BPPV patients may be complicated by cerebrovascular diseases, resulting in the vertebral artery dominance and the abnormality of the basilar artery, which could be diagnosed by imaging features of MRI. To sum up, it was of great significance for the clinical diagnosis and treatment of BPPV patients to explore the role of the vertebrobasilar artery on BPPV patients, and to find effective methods to delay the disadvantages caused by vertebral artery dominance and abnormal basilar artery [20]. Among the 60 patients, 20 cases with CMBs were detected by SWI, while 40 cases of CMBs were detected by conventional MRI scanning. It was proved that the SWI examination under the PDDC-CNN model had an excellent performance in diagnosing cerebral hemorrhage.

## 5. Conclusion

The artificial intelligence algorithm under PDDC-CNN was successfully applied. It was combined with conventional MRI scanning and MRA to explore the correlation between intracranial small blood vessels as well as vertebrobasilar arteries and BPPV in China. It was discovered that the MRI features of BPPV patients were in a close relation to their own microvascular lesions, and it was speculated that cerebrovascular factors might be dominant in the early onset of BPPV. Deep learning-based MRI could diagnose symptoms such as vertebral artery dominance and basilar artery abnormality more accurately in patients with BPPV, so it was worth being popularized clinically. However, the samples were relatively less, and the relationship between the location of CMBs, the abnormality of the vertebrobasilar artery and its branches, and the degree of BPPV had not been explored. The correlation and mechanism between the abnormalities of the basilar artery and its branches and the degree of BBPV could be investigated in the future with more samples and deeper methods. All in all, the results offered data reference for the imaging diagnosis of BPPV patients as well as the prevention and treatment of cerebrovascular diseases.

## Data Availability

The data used to support the findings of this study are available from the corresponding author upon request.
